# Attitudes and knowledge of myopia management by Spanish optometrists

**DOI:** 10.1007/s10792-023-02835-7

**Published:** 2023-08-18

**Authors:** Johnny Di Pierdomenico, Raquel González-González, Francisco J. Valiente-Soriano, Caridad Galindo-Romero, Diego García-Ayuso

**Affiliations:** 1 Facultad de Óptica y Optometría, Universidad de Murcia, Murcia, Spain; 2 Grupo de Investigación Oftalmología Experimental, Departamento de Oftalmología, Optometría, Otorrinolaringología y Anatomía Patológica, Facultad de Medicina, Universidad de Murcia, Instituto Murciano de Investigación Biosanitaria (IMIB), Campus de Ciencias de la Salud, 30120 Murcia, España

**Keywords:** Myopia control, Optometry, Orthokeratology, Dual-focus soft contact lens, Atropine

## Abstract

**Purpose:**

To investigate the knowledge, training and clinical practice of Spanish optometrists about preventing and controlling myopia progression.

**Methods:**

A web-based questionnaire was distributed to Spanish optometrists through social networks, optometric professional bodies and one of the major Spanish optometrists' associations to assess practitioner perception, understanding, and self-reported clinical practice behavior related to myopia diagnosis and management.

**Results:**

A total of 534 optometrists with a mean age of 40.8 ± 10.3 years completed the survey. Most respondents have been practicing optometry for more than 20 years (89.8%), report having actively treated childhood myopia (82.4%), and are very concerned about the increasing frequency of pediatric myopia in their daily practice (85.3%). Almost all of the respondents (97.3%) agreed that the efficacy of treatment is related to the age at which it is prescribed, and more than half (53.6%) considered a progression higher than − 0.50 and up to − 1.00D as the minimum necessary to consider a myopia management option. Respondents who reported actively managing childhood myopia considered orthokeratology, atropine and soft-defocus contact lenses the most effective myopia control interventions. However, the most frequently prescribed form of myopia correction by Spanish optometrists was single-vision spectacles, followed by orthokeratology and soft-defocus contact lenses.

**Conclusions:**

Spanish optometrists are very active in the management of myopia, especially by fitting orthokeratology lenses or dual-focus soft contact lenses for myopia control, but there is still potential for improvement in the methodology they follow for both the diagnosis and management of myopia.

**Supplementary Information:**

The online version contains supplementary material available at 10.1007/s10792-023-02835-7.

## Introduction

Myopia is a refractive error defined as an equivalent spherical refraction ≤  − 0.50 diopter (D) [1]. It affects almost 30% of the world's population [2], with a rising prevalence. Between 2010 and 2020, the prevalence of myopia worldwide has increased by 20% [2, 3]. In Spain, the prevalence of myopia increased between 2016 and 2020, reaching 20.4% [4]. Projections estimate that by 2040 half of the population will be myopic [2]. Increasing the risk of secondary complications of high myopia and, thus, its burden and social cost [5, 6]. Uncorrected refractive errors are one of the leading causes of preventable blindness in the world [3]. Yet, myopia could become one of the leading causes of irreversible blindness in the world.

High myopia is defined as an equivalent spherical refraction ≤  − 6.00 D or an axial length exceeding 26 mm [1]. Above this level of myopia, the risk of uncorrectable vision loss increases [1], and this risk increases as myopia rises [7]. It is important to emphasize that there is no safe level of myopia and that each diopter aggravates its consequences [7].Thus, interventions should not only aim at preventing high myopia but also at preventing the onset and progression of low myopia. Indeed, there is increasing interest in the development of strategies aimed at preventing and/or slowing the onset and progression of myopia and axial length growth [8], the main modifiable risk factor for the pathological consequences of myopia, such as myopic maculopathy [9]. There are currently several interventions to prevent the development of myopia, such as increasing the amount of time spent outdoors. And also, to slow its progression, including pharmacological and optical measures, some of them used off-label [8]. But, to date, there is no global consensus on guidelines for standardized management of myopia [10, 11]. Different professions (optometrists, ophthalmologists and dispensing opticians) and countries applied different strategies [12].

Clinical management of myopia progression is new, and the scientific evidence is evolving. Thus, it is necessary to better understand optometrist myopia knowledge and clinical practices. To the best of our knowledge, only a few studies have examined this fact in depth to date [12–15]. Only one of them specifically analyzed data from Spanish optometrists [14].But the data in this earlier study came from an international survey, thus covering a much larger sample size [12, 14]. This international questionnaire was not specifically designed for Spanish optometrists [14].These studies highlighted the need to establish clinical guidelines for myopia management [13]. Spanish optometrists are not allowed to use diagnostic (e.g., cycloplegic) or therapeutic (e.g., atropine) eye drops, so we consider it of interest to know their involvement in the active control of myopia progression given these limitations. This study aims to investigate Spanish optometrists' knowledge of myopia. But also, their training and clinical practice using a self-reported survey. And, finally, to understand training needs and the evidence on which they base their clinical practice.

## Methods

An online survey hosted on Google Forms (Google Inc., CA, USA) was developed and distributed to Spanish optometrists through social networks, optometric professional bodies and one of the major Spanish optometrists' associations, AEOptometristas. Participation in the study was voluntary, and consent was implied by submission of the questionnaire. The study received ethics approval from the Ethics Committee of the University of Murcia.

### Survey design

The questionnaire was developed in Spanish based on a previously published work [13] with slight modifications (Online Appendix 1). The survey consisted of 35 questions and was divided into three different sections: (1) demographic data of the respondents, (2) perception, understanding and self-reported behavior of the practitioner in clinical practice in relation to myopia diagnosis and management, and (3) questions to those practitioners who do not manage myopia.

In the questionnaire, myopia was defined, following the International Myopia Institute (IMI) guidelines [1], as a spherical equivalent refractive error ≤  − 0.50 D in the absence of accommodation. Most of the questions, except those requiring a written answer, were presented in such a way that the participant had to choose the answer from a list of possibilities. Each participant was asked to indicate whether he/she was an optometrist at the start of the survey, and only those who indicated that they were optometrists were allowed to continue completing the survey. In the questionnaire, the authors decided to distinguish between Diploma and Bachelor's Degree, since Bacherlor's Degree appeared because of the European Higher Education Area, which meant that optometric training in Spain was more oriented toward clinical activity and health sciences since then. However, the professional competencies, according to Spanish law, are the same for both degrees. It is important to note that, although the pharmacological management of myopia was also questioned, Spanish optometrists are healthcare professionals who can prescribe optical correction but, as previously mentioned, cannot prescribe drugs unless they work with an ophthalmologist.

### Statistical analysis

Only completed surveys by optometrists were used in the analyses. Statistical analyses were performed using the Statistical Package for Social Sciences software version 24 (SPSS, International Business Machine Corp. IBM, Chicago, IL, USA). Univariate analyses (Chi-square test) were performed to detect significant differences between different variables (active management of childhood myopia, higher concern about the link between myopia progression and related ocular pathologies, optometric practice experience, use of treatments for myopia control, educational qualification (academic degree), city/town population, and place of optometric practice). A multivariate binary logistic analysis model was performed to answer the question of whether optometrists use treatments for myopia control considering optometric practice experience, educational qualification (academic degree), city/town population, the actual place of optometric practice, number of myopic patients under 16 years of age attended to in a typical week, and concerns about the increasing frequency of pediatric myopia as covariates. The level of significance was set at *P* < 0.05.

## Results

### Demographics

A total of 534 completed surveys were received, representing 3% of the total number of 18,271 optometrists registered in Spain according to the Spanish National Statistics Institute in January 2023. The mean age of respondents was 40.8 ± 10.3 years with a range of 21–69 years. Most respondents had been practicing optometry for more than 20 years (89.8%) and slightly less than half (43.5%) had a master’s degree. The demographics of the respondents are shown in Table 1.

**Table 1:** Demographics of the participants

Characteristics	Answers (*n* = 534)
Gender, female/male: *n* (%)	354/180 (66.3%/33.7%)
Age	40.8 ± 10.3
Degree: *n* (%)	
Diploma in optics and optometry	200 (37.4%)
Bachelor's degree in optics and optometry	98 (18.4%)
Master's degree in clinical optometry or similar	232 (43.5%)
PhD	4 (0.7%)
Optometric practice experience: *n* (%)	
≤ 5 years	84 (15.7%)
6–10 years	68 (12.7%)
11–15 years	80 (15%)
16–20 years	62 (11.6%)
> 20 years	240 (89.8%)
Place of optometric practice: *n* (%)	
Independent	172 (32.2%)
Corporate	280 (52.4%)
Ophthalmology clinic or public hospital	82 (15.4%)
City/town population: *n* (%)	
< 10,000	36 (6.7%)
10,001–20,000	76 (14.2%)
20,001–100,000	150 (28.1%)
100,001–500,000	160 (30%)
> 500,001	82 (15.4%)
Don´t know	30 (5.6%)
Number of myopic patients under 16 years of age attended to in a typical week: *n* (%)	
≤ 5 patients	206 (38.6%)
6–10 patients	174 (32.6%)
11–15 patients	64 (12%)
16–20 patients	26 (4.9%)
> 20 patients	64 (12%)

### Myopia diagnosis

Participants were asked to indicate the number of myopic patients under the age of 16 that they usually see in a typical week and 61.4% (328) indicated that they provided clinical care to more than 5 myopic children per week. They were also asked to indicate on a scale of 1 (no concern) to 10 (extremely concerned) their level of concern about the increasing frequency of pediatric myopia (onset between 5 and 16 years) resulting in a mean score of 8.6/10, with 85.3% (456) of participants indicating a score of at least 8/10 (very concerned), mainly because of the possible link between myopia progression and related ocular pathologies (78.2%; 418).

Subsequently, a list of clinical procedures was presented to participants, and they were asked to select those that they routinely perform at the first visit of a school-age myope. More than 90% of respondents indicated that they note a family history of myopia (94.8%; 506) and undertake a non-cycloplegic subjective refraction (95.5%; 510), whereas only 14.2% (76) undertake cycloplegic refraction. Other frequent measurements were cover test (74.9%; 400), non-cycloplegic autorefraction (81.6%; 436) and non-cycloplegic retinoscopy (69.3%; 370). Cycloplegic subjective refraction (14.2%; 76) and retinoscopy (5.2%; 28) were infrequent, as were other interventions summarized in Table 2.

**Table 2:** Optometrists’ responses relating to the clinical exams routinely performed at the first visit of a school-age myope (5–16 years)

Clinical exams	*n* (%)
Non-cycloplegic subjective refraction	510 (95.5%)
Family history of myopia	506 (94.8%)
Non-cycloplegic autorefraction	436 (81.6%)
Cover test	800 (74.9%)
Non-cycloplegic retinoscopy	370 (69.3%)
Stereopsis	298 (55.8%)
Corneal topography	184 (34.5%)
Intraocular pressure	180 (33.7)
AC/A ratio	162 (30.3%)
Dynamic retinoscopy	152 (28.5)
Pupil size measurement	130 (24.3%)
Undilated retinal fundus examination	124 (23.2%)
Retinography	98 (18.4%)
Cycloplegic subjective refraction	76 (14.2%)
Ocular axial length measurement	72 (13.5%)
Dilated retinal fundus examination	48 (9%)
Optical coherence tomography (OCT)	42 (7.9%)
Cycloplegic retinoscopy	28 (5.2%)
Accommodative flexibility	12 (2.7%)
Ocular motility	10 (1.8%)

Participants were then asked if they applied any type of treatment for myopia control, and 82.4% (440) stated that they did. Of them, only 31.8% (140) declared providing myopia care for more than five years, 30.9% (136) provide it for less than two years, and 63.6% (280) reported being very active in myopia management in their clinical practice. These 440 respondents continued to complete the remainder of the questionnaire.

### Perceived efficacy of myopia control and preventive interventions

The 440 respondents who continued with the questionnaire were asked to choose from a list, the treatment they considered most effective in controlling the progression of myopia. More than half of them considered orthokeratology (55.5%; 244) the most effective myopia control intervention, followed by atropine (17.7%; 78) and soft defocus contact lenses (10.9%; 48) (Table 3).

**Table 3:** Optometrists’ responses relating to the consideration of the most effective myopia control treatment for a patient in a school-age myope (5–16 years)

Treatment	*n* (%)
Orthokeratology	244 (55.5%)
Atropine (no differentiation of concentrations)	78 (17.7%)
Soft defocus contact lenses (myopia control)	48 (10.9%)
Spectacles lenses for myopia control	12 (2.2%)
Visual hygiene	16 (3.6%)
Combination of Orthokeratology and Atropine	16 (3.6%)
Increase time spent outdoors	6 (1.4%)
Spectacle lenses (under correction)	4 (0.7)
Soft contact lenses (full correction)	4 (0.7)
Progressive addition spectacle lenses	0 (0%)
Bifocal spectacles lenses	0 (0%)
Spectacle lenses (full correction)	0 (0%)
Bifocal soft contact lenses	0 (0%)
RGP contact lenses	0 (0%)
Bifocal RGP contact lenses	0 (0%)
Multifocal RGP contact lenses	0 (0%)

Almost all the respondents (97.3%; 428) agreed that the efficacy of the treatment is related to the age at which it is prescribed. Only 37.2% (164) considered that a progression of up to − 0.50D required active management, whereas more than half (53.6%; 236) considered a progression higher than − 0.50D and up to − 1.00D to be necessary.

When surveyed about the recommendations they usually give to their patients under 16 years old to reduce the likelihood of myopia onset and progression, practitioners indicated they recommend increasing the time they spend outdoors (93.6%; 412), maintaining an adequate reading distance (85.9%; 378), decreasing screen (80%; 352) and smartphone (79.5%; 350) usage time and reading in daylight conditions (70%; 308).

### Current myopia management practices

**Fig. 1:**
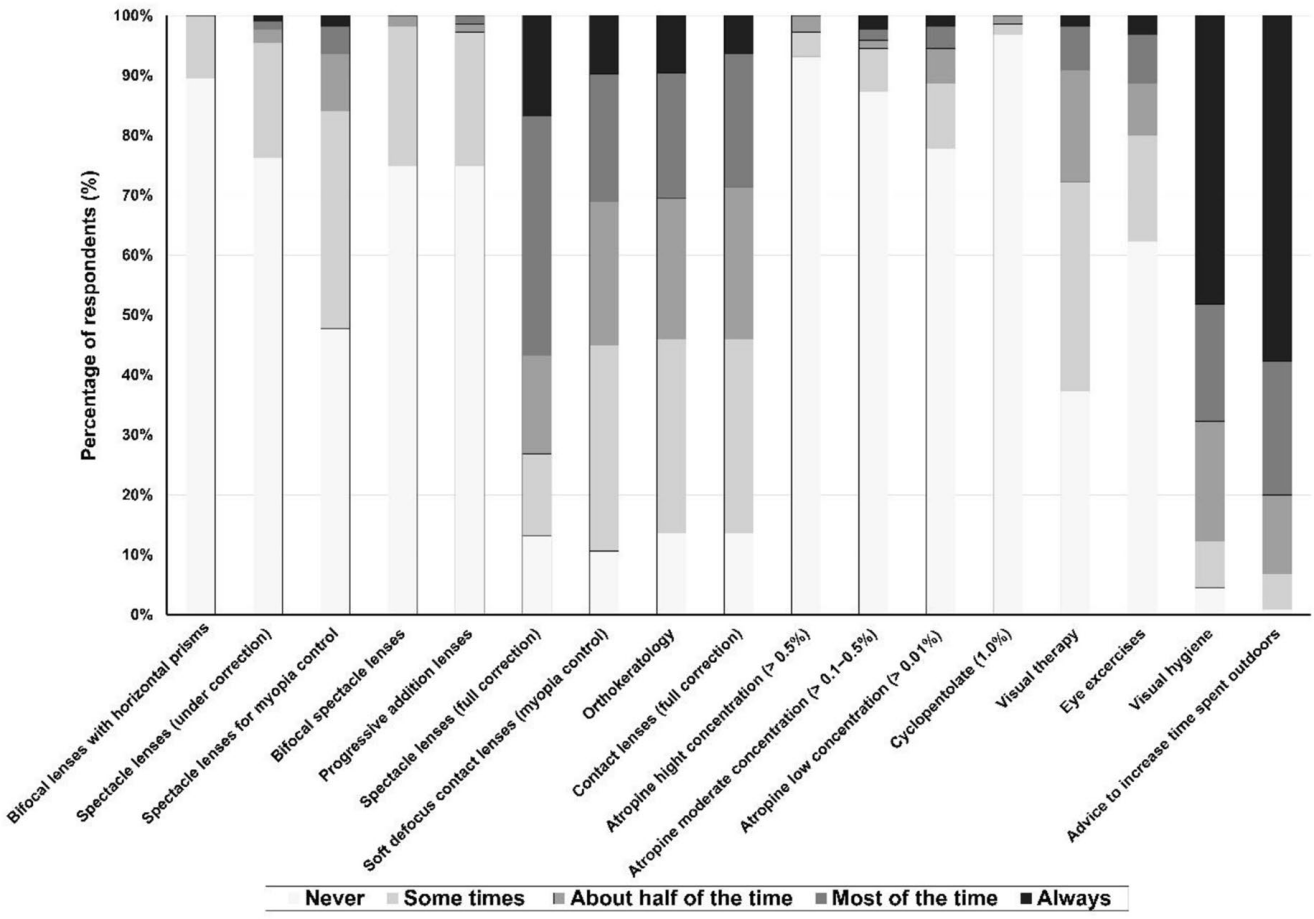
Frequency of prescription of different strategies for myopia control

The most frequently prescribed forms of myopia correction were single-vision spectacles, orthokeratology and soft defocus contact lenses, with 57% (250), 33.5% (134) and 30.4% (134) of respondents, respectively, indicating that they prescribed these modalities “most of the time” or “always” (Fig. 1). Atropine and multifocal, bifocal, or spectacles lenses for myopia control were rarely prescribed. Of these treatments, spectacle lenses for myopia control and low-dose atropine were the most frequently used, being prescribed at least “about half of the time” by about 16% (70) and 11.4% (50), respectively (Fig. 1).

**Fig. 2:**
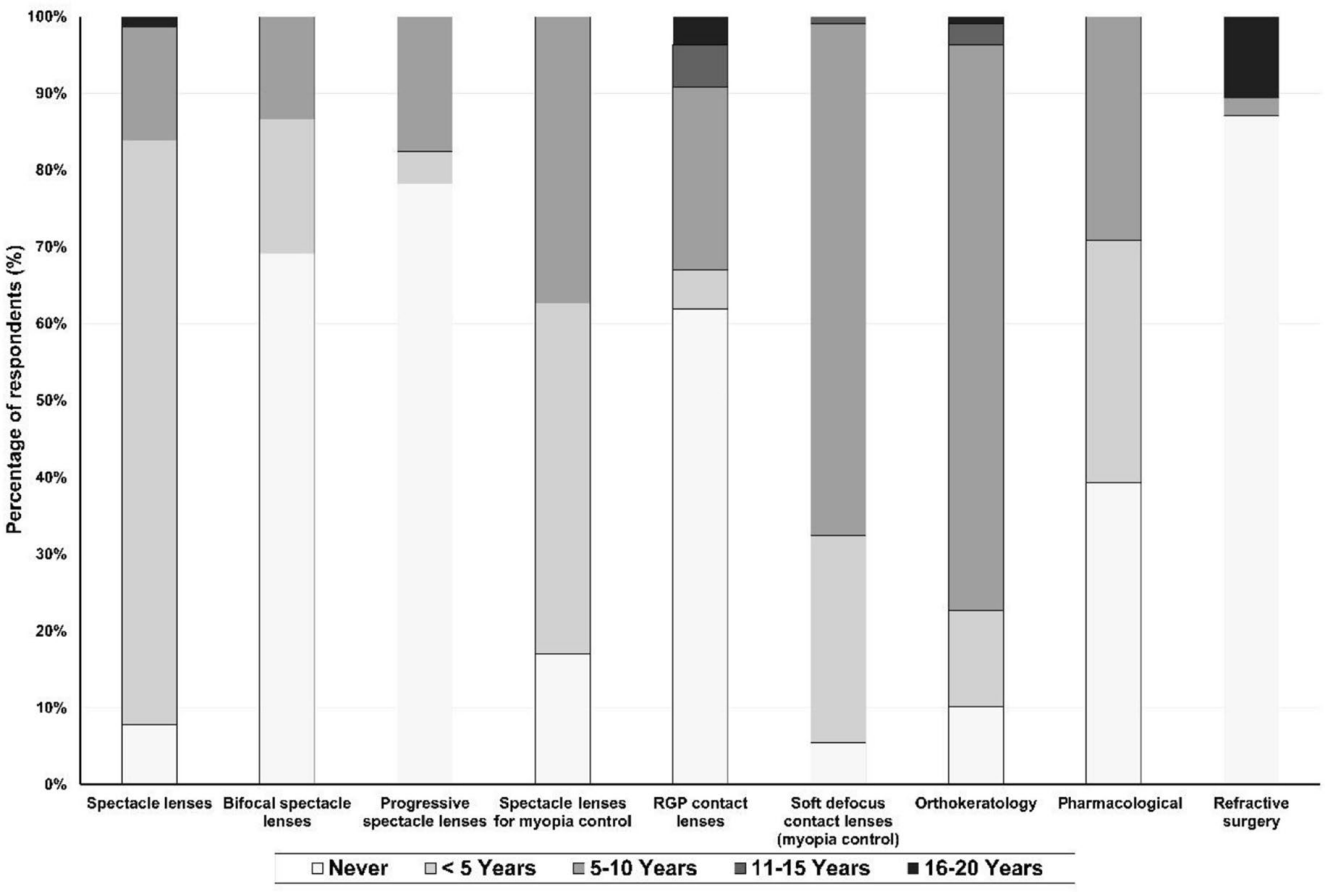
Most prescribed forms of myopia correction according to children age

Considering the age at which myopia is detected, the most prescribed treatment for a child under the age of 5 was single-vision spectacles with 75% (330) of respondents followed by spectacle lenses for myopia control with 45% (198) (Fig. 2). Regarding contact lens options, only 26.8% (118) and 12.3% (54) reported prescribing soft defocus contact lenses and orthokeratology, respectively (Fig. 2). Thirty-point nine percent (136) stated that they recommended atropine for a child under the age of 5 (Fig. 2).

**Fig. 3:**
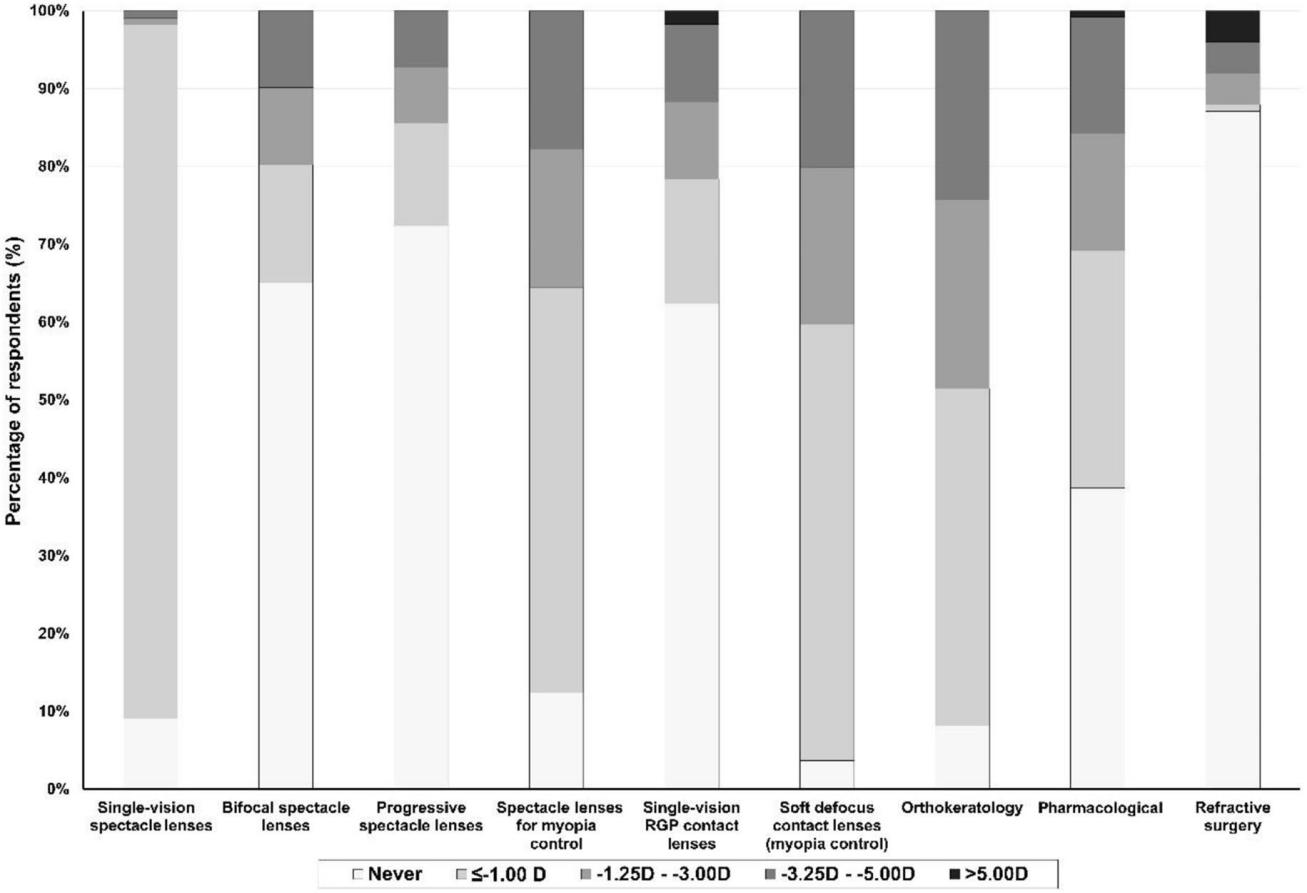
Most prescribed forms of myopia correction according to myopia levels

Regarding myopia levels, for patients with low levels of myopia (≥ − 1.00D) single-vision spectacles (89.5%; 394), soft defocus contact lenses (69.5%; 306), spectacle lenses for myopia control (62.3%; 274) and orthokeratology (55.9%; 246) were the most prescribed options (Fig. 3), while for levels of myopia <  − 1.00D single-vision spectacles were infrequently prescribed (Fig. 3).

**Fig. 4:**
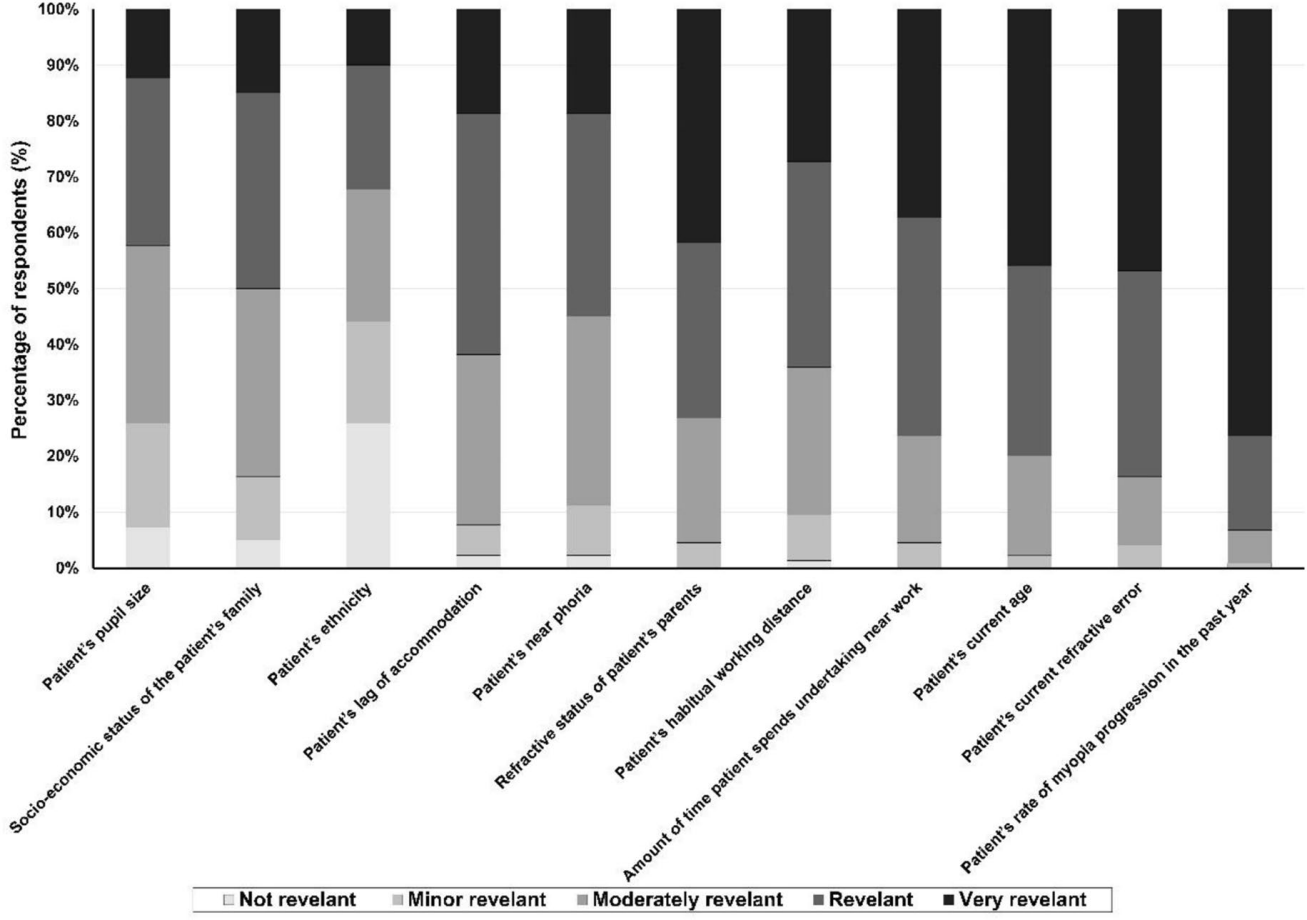
Importance of different factors in deciding on a myopia management strategy

Respondents were then asked about the importance they attributed to several factors when deciding on a myopia management strategy for a pediatric patient (Fig. 4). The rate of myopia progression in the last year was the key factor identified as “very important” by most of the respondents (76.3%; 336). Child current refractive error (83%; 368), patient age (80%; 352), amount of time spent in near work (75.9%; 334) and parents’ refractive error (72.7%; 320) were considered “important” or “very important”. Factors considered to be less important were the patient´s ethnicity (32.2%; 298), pupil size (42.2%; 254) and socio-economic situation of the family (50%; 220).

Participants were then asked to indicate whether pharmacological and/or optometric treatments for myopia control were applied at their workplace.

Only 11.4% (50) of the respondents reported working where pharmacological management of myopia progression is applied. Subsequently, these 50 participants were asked about the earliest age at which they usually applied pharmacological treatment, with the mean age being 4.6 ± 1.8 years and 80% ranging from 4 to 8 years of age. The most commonly used pharmacological myopia management option for these 50 optometrists (Table 4) is low-dose (0.01%) atropine eye drops (92%). Only 36% of the respondents indicated that they had some patients who had discontinued pharmacological management of myopia and, of these, 44.4% reported a rebound effect.

**Table 4:** Optometrists’ responses relating to the type of treatment they commonly use as myopia control

Optometric treatment (*n* = 426)	*n* (%)
Soft defocus contact lenses (myopia control)	312 (73.2%)
Orthokeratology	312 (73.2%)
Spectacle lenses (full correction)	144 (33.8%)
Spectacles lenses for myopia control	134 (31.5%)
Soft contact lenses (full correction)	130 (30.5%)
RGP contact lenses	34 (8%)
Progressive addition spectacle lenses	34 (8%)
Spectacle lenses (under correction)	34 (8%)
Bifocal spectacles lenses	22 (5.2%)
Bifocal soft contact lenses	8 (1.9%)
Multifocal RGP contact lenses	8 (1.9%)
Bifocal RGP contact lenses	0 (0%)

Pharmacological treatment in collaboration with an ophthalmologist (*n* = 50)	*n* (%)
Atropine 0.01%	46 (92%)
Atropine 0.5%	10 (20%)
Atropine 0.02%	2 (4%)
Atropine 0.05%	2 (4%)
Atropine 1%	0 (0%)
Pirenzepine	0 (0%)
Cyclopentolate	0 (0%)
Tropicamide	0 (0%)
Timolol	0 (0%)

When asked about the type of myopia management performed in their workplace, most participants who were active in myopia treatment (96.8%; 426) stated that they worked in a center where optometric myopia treatment was provided. Participants were asked to indicate the youngest age at which they usually recommend these optometric treatments for myopia control, with a mean minimum age of 6.3 ± 1.9 years, where 74.7% of the respondents were within the range of 4–8 years of age. The most commonly used myopia management options for these respondents (Table 4; more than one option was allowed to be selected if deemed necessary) were orthokeratology (73.2%; 312) and soft defocus contact lenses (73.2%; 312), followed by spectacle lenses for myopia control (31.5%; 134). However, even among optometrists working in centers where optometric treatments for myopia control were applied, the use of traditional myopia correction techniques was still relatively common, with 30.5% (130) and 33.8% (144) of respondents indicating that they frequently recommended single-vision distance (full correction), soft contact lenses (30.5%; 130) or spectacles (33.8%; 144), respectively. Participants were then asked about their experience with patients who discontinued optometric treatment for myopia control and whether they observed any rebound effect, with less than half (41.3%; 176) of the participants, indicating that they had had this experience and, of these, only 35.2% (62) had observed a rebound effect.

### Barriers to myopia management practices

**Fig. 5:**
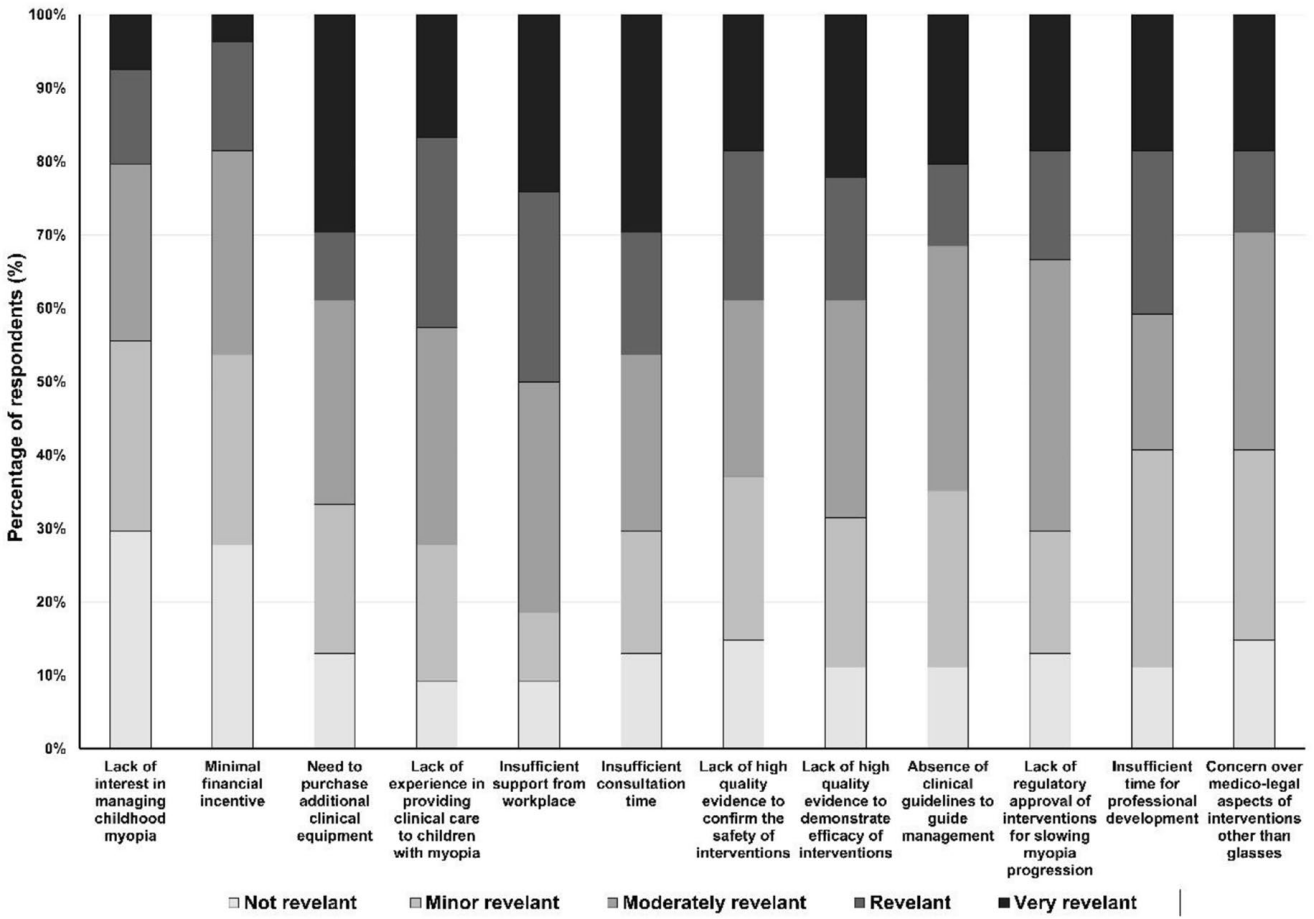
The relative importance of factors limiting optometrists' ability to provide optimal clinical care for children with myopia

Only 17.6% (94) of the respondents reported not managing childhood myopia, and 3.2% (14) of those who manage childhood myopia declared not applying optometric management of myopia, amounting to a total of 20% (108) of the respondents. These optometrists were questioned about the relative importance of certain factors in limiting their ability to provide optimal clinical care for children with myopia. The need to purchase additional clinical equipment and the lack of time in the optometric practice were the key factors identified as “very important” by respondents (29.6% each). Lack of support in the workplace was cited as the main constraint since 50% (54) considered this factor as important or very important. Other factors considered as important or very important were lack of time in the optometric practice, lack of experience, and lack of time for professional training identified by 46.3% (50), 42.6% (46), 40.7% (44), respectively. Lack of professional interest and financial incentives were considered "not important" or “minor” by 55.5% (60) and 53.7% (58), respectively. Other factors such as lack of evidence, lack of clinical guidelines, or lack of regulatory approval of interventions were considered "moderately important" (Fig. 5). Optometrists were also asked to indicate the reasons that had prevented them from prescribing an alternative method to single-vision glasses or contact lenses for myopia (perform myopia control) by providing a list of reasons from which they were allowed to select more than one. Respondents indicated aspects such as cost to the patient (51.9%), lack of information/knowledge (40.7%), or limitations in their workplace (35.2%), among others (Table 5).

**Table 5:** Optometrists’ responses relating to the reason that has prevented to use other treatments for myopia control if they have only adapted monofocal spectacles/contact lenses to myopic patients

Motivation	*n* (%)
The treatment price to the patient	56 (51.9%)
Lack of experience in providing clinical care to children with myopia	44 (40.7%)
I am not allowed to perform this type of treatment in the work center	38 (35.2%)
Additional work time required for the professional	26 (24.1%)
Results are not predictable	18 (16.7%)
I do not believe that these treatments are effective	12 (11.1%)
Lack of high-quality evidence to confirm the safety of interventions	10 (9.3%)
Risk/benefit ratio	10 (9.3%)
Not enough patients with the required conditions to use other treatments	2 (3.8%)

### Univariate analysis

There was a significant relationship between the respondents who considered themselves more active in managing childhood myopia and those who reported a higher concern about the link between myopia progression and related ocular pathologies (*χ*^2^(12) = 52.720, *P* < 0.05). Regarding optometric practice experience, those reporting an older experience in the profession were more likely to use treatments for myopia control (*χ*^2^(4) = 16.514, *P* < 0.05). Optometrists working in bigger cities are more likely to use treatments for myopia control (*χ*^2^(5) = 25.993, *P* < 0.001). Optometrists working in private practices (independent or corporate) are more likely to use treatments for myopia control than those working in an ophthalmology clinic or public hospital (*χ*^2^(1) = 12.247, *P* < 0.001). However, there was no link between a higher academic degree and more active management of childhood myopia (*χ*^2^(3) = 5.442, *P* = 0.142).

### Multivariate analysis

Multivariate binary logistic analysis showed optometric practice experience (*P* = 0.0375) and educational qualification (academic degree) (*P* = 0.0233) as the only statistically significant variables with respect to the use or not of treatments for myopia control. The respective odds ratios (95% CI) were 1.3275007 (1.02000645–1.746529) for optometric practice experience and 1.8483109 (1.11275737–3.246454) for educational qualification (academic degree).

## Discussion

The global burden of myopia [16] and its related complications [3, 5] is increasing worldwide [11], which has led to an increased interest in the study of myopia and its management strategies challenging optometrists around the world with a new scenario for treating myopic children, due to the continuous emergence of new scientific evidence concerning the prevention and control of myopia progression [8], as well as the need for additional training for its correct application. The main objective of this work was to assess the opinion and clinical practice of Spanish optometrists through an online survey.

According to this study, most Spanish optometrists are concerned about the increasing frequency of childhood myopia and its related ocular pathologies [14]. Indeed, more than half of the respondents reported providing clinical care for at least 6 myopic children per week, which is higher than the percentage found in another recent study in Australia, in which more than half of the participants reported providing clinical care to 5 or less myopic children per week [13]. Almost all respondents indicated that they routinely note the family history of myopia during the baseline visit, in accordance with previously published data [13]. This fact is important since myopia may have an important genetic background [17–20] that may interact with environmental determinants [18, 20–23]. Most respondents reported performing retinoscopy, a gold standard test for refractive error, and non-cycloplegic subjective refraction which while it might not be the most appropriate for a myopic child [24], since non-cycloplegic subjective refraction and autorefraction may overestimate myopia [25], would be a correct way if accommodation is properly relaxed during subjective refraction [10, 26].It is important to note that in Spain optometrists are not authorized by law to administer drugs like cycloplegic drops. Most participants did not routinely perform binocular vision assessment and this is an important aspect, although there is still no consensus on the gold standard techniques to be used [10]. In fact, only 28% and 30% reported performing dynamic retinoscopy and calculating the AC/A (accommodative-convergence to accommodation) ratio, respectively, although the vast majority did routinely perform a cover test. Ocular accommodation and near work are not factors that cause the onset of myopia [27]. However, they are associated with its development and progression [27]. An increasing AC/A ratio, a clinical method to measure impairments of binocular vision, is related to a greater lag of accommodation and has been proposed as an early sign of becoming myopic [28]. Indeed, myopic children are more likely to have a higher AC/A ratio [27, 28], which usually peaks at myopia onset [28], and a higher lag of accommodation [28, 29], which causes retinal defocus at near work [27]. This retinal defocus is thought to be the final trigger of axial elongation [30]. It would therefore be advisable for all optometrists providing clinical care to children to calculate the AC/A ratio as binocular vision is key in the formation of the retinal image. Surprisingly, very few respondents indicated performing ocular biometry routinely although monitoring eye growth is considered an important test, since longest axial length is related to higher myopia [26] and the risk of its associated pathologies. However, the percentage of respondents that reported performing ocular biometry in this study (13.5%) is higher than that found in another similar study (3%) [13]. This finding may be due to the difficulties that optometrists and, in particular, optical retailers may encounter in accessing relatively expensive equipment such as biometers [13].

Most respondents reported that the efficacy of the treatments relies on the age of the children and that the earliest age at which management is most frequently applied is between 4 and 8 years of age. It is important since younger age is one of the main factors associated with a faster myopic progression and a greater likelihood of high myopia in adulthood [31], together with female sex and ethnicity [32]. More than half of the respondents declared that an increase of more than − 0.50 D per year is necessary to begin myopia management, in accordance with a previous work in Spain [14]. It has been shown that each diopter increase in myopia increases the risk of developing myopic maculopathy by 67% and that there is no safe level of myopia [7], so it seems necessary to reinforce the information that reaches optometrists in this respect. Regarding the most effective treatments for slowing myopia progression, respondents reported orthokeratology (55.5%), atropine (17.7%) and soft defocus contact lenses (10.9%), which is in accordance with a previous report from Spanish optometrists [14]. Orthokeratology is considered one of the most effective optical treatments for myopia control, even in the long-term, with a reported efficacy in slowing axial length from 32 to 63% [33]. Antimuscarinic topical medications such as atropine are also effective but lead to adverse effects [33].There are still many unanswered questions about these treatments, such as what happens when they are interrupted or how long they should be maintained. On the other hand, combination treatments such as spectacles or contact lenses plus atropine are still in their infancy and need to be further studied until sufficient evidence is generated to be translated into clinical practice [34]. Currently, soft defocus contact lenses are steadily becoming more popular in countries such as Spain thanks to the evidence that has recently been generated about their effectiveness [14, 35]. Interestingly, although orthokeratology is considered to be the most efficacious intervention by far, it is applied by Spanish optometrists to myopic children in the same percentage of fitting as soft defocus contact lenses. Because in Spain pharmacological treatments cannot be prescribed by optometrists but must be administered by an ophthalmologist, participants were asked if they worked with an ophthalmologist in a clinic where drugs were administered and only answered questions about the administration of atropine if this was the case. If not, they responded directly to the questions about optometric treatment questions. Low-dose atropine (0.01%) is by far the most applied pharmacological intervention, but only in clinics where the treatment is administered in collaboration with an ophthalmologist. Regarding traditional myopia compensation methods for children, according to this study, currently, the most commonly prescribed in Spain is still single-vision spectacles, although there is no proven benefit in terms of controlling the progression of myopia [36]. In fact, it is the most frequently prescribed form of myopia correction not only by Spanish optometrists [14] but also worldwide [12], despite being considered ineffective for the management of myopia progression.

Survey respondents declared that their advice to their patients under the age of 16 regarding myopia onset and progression included the need to spend more time outdoors, maintain an adequate reading distance and reduce exposure time to all types of electronic devices. Near work activities and, in particular, those involving electronic devices have been proposed to be related to a higher myopia incidence [37, 38]. The amount of time children spend outdoors is a significant risk factor for myopia onset [19, 37–40] together with parental history of myopia [19]. However, it is somewhat unclear whether time outdoors could reduce myopia progression once it has already appeared [39]. In these last years, the increased time in near work with digital devices [41, 42] together with the reduced time spent outdoors due to the COVID-19 pandemic may have increased the risk of myopia onset and progression in children [43, 44], a side effect of the measures to contain the pandemic that will only become apparent in the next few years [45, 46]. Interestingly, recent works have proposed that time spent outdoors may have also had a role in the efficacy of dual-focus soft contact lenses for myopia control [47] and atropine [48, 49] during the pandemic, while another recent study has proposed that dual-focus soft contact lenses for myopia control are effective regardless of time spent outdoors [50]. Another recent work has proposed a stronger association between digital screen use and myopia progression than between myopia and time spent outdoors during the strict lockdown [45]. Therefore, the complex interaction [20] between time outdoors, near work and myopia onset and progression needs further investigation. Finally, the barriers identified by Spanish optometrists who do not practice myopia management include the need to purchase additional clinical equipment, the lack of time in the optometric practice, the cost for the patient and the lack of time for professional training in accordance with similar studies performed in Australia [13] and Spain [14], suggesting that the overall gaps for incorporating myopia management into daily practice are very similar.

To our knowledge, only one study to date has analyzed myopia control strategies in Spanish optometrists. However, this study was not specific, as the data were obtained from an international survey consisting of only 9 questions [14]. Moreover, the sample of participants in the present study (534) is much larger than the sample obtained in the previous study (173) [14]. Both studies agree that single-vision spectacles and contact lenses remain the most commonly used method of myopia correction [14], except in the case of optometrists working in centers where active myopia control is performed, with this distinction being made only in the present study. Both studies also agree on the fact that Spanish optometrists consider orthokeratology to be the most effective myopia control method [14]. However, in the present study there is an upward trend for multifocal contact lenses for myopia control, which may be because the referred survey was conducted before (October 2018–April 2019), and there is increasing evidence for the efficacy of these contact lenses [51, 52]. Surprisingly, both studies also agree that a relatively high percentage of Spanish optometrists still consider outdoor time as an option for effective control of myopia progression [14], contrary to current evidence suggesting its role in controlling the onset but not the progression of myopia [8, 39, 53] (see above). Finally, both studies agree financial cost to the patient is one of the main limiting factors to provide a myopia control strategy [14].

This study has some limitations. One of the main limitations could be the number of participants who responded to the survey. However, although the response rate has not been as high as would have been desirable, it is important to note that it is higher than would have been necessary to achieve a confidence level of 95% with a margin of error of 5% (377). Another limitation may be related to the distribution by electronic sources. While it is true that the electronic distribution of the questionnaire could be biased as it would limit the response to optometrists who are computer and social network literate, it is also true that it allows access to a larger population and a greater geographical diversity. Finally, because this was a voluntary survey, it is possible that optometrists who are more supportive of myopia management and control were more likely to respond to the survey, which could bias the results toward an overestimation of proactive myopia promotion.

In summary, this work reflects the knowledge and clinical practices of Spanish optometrists. The study shows that Spanish optometrists are concerned about the increasing incidence of myopia in their clinical practice and that they are willing to do something about it. However, it also highlights the need to be able to perform refractions under cycloplegia, which is currently impossible in an optical establishment in Spain, as well as the need for optical establishments to have biometers or other devices to measure axial length. Spanish optometrists are very active in the management of myopia, especially by fitting orthokeratology lenses and dual-focus soft contact lenses for myopia control, but there is still room for improvement in the methodology they follow for both the diagnosis and management of myopia. It is also important to stress the need for the optometrist to collaborate with the ophthalmologist in the management of myopia, especially in countries like Spain where the optometrist does not have access to certain treatments, such as pharmacological treatments. The availability of further evidence on myopia management, as well as new policy regulations, should enable optometrists around the world to increase their contribution to this global health problem.

### Supplementary Information

Below is the link to the electronic supplementary material.

Supplementary file1 (DOCX 62 KB)
