# Supplementary material for: Attitudes and knowledge of myopia management by Spanish optometrists
Source: Int Ophthalmol. 2023 Aug 18;43(11):4247–61. doi: 10.1007/s10792-023-02835-7 (PMC10520101; doi:10.1007/s10792-023-02835-7)
Supplement: Supplementary file 1 — Supplementary file1 (DOCX 62 KB) [file 10792_2023_2835_MOESM1_ESM.docx]

**Appendix 1. Questionnaire**

**I: General information**

1. Age: …………………

2. Gender:

1. Male
2. Female

3. Are you an optometrist working in Spain?

1. Yes
2. No

4. Indicate your highest degree:

1. Diploma in Optics and Optometry
2. Bachelor's Degree in Optics and Optometry
3. Master's Degree in Clinical Optometry or similar
4. PhD

5. Indicate your experience in the practice of optometry:

1. ≤ 5 years
2. 6-10 years
3. 11-15 years
4. 16-20 years
5. > 20 years

6. Indicate your current place of optometric practice:

1. Independent
2. Corporate
3. Ophthalmology clinic or public hospital

7. How many inhabitants live in the town or city where you practice?

1. ≤ 10,000
2. 10,001 -20,000
3. 20,001-100,000
4. 100,001-500,000
5. > 500,001
6. Don’t know

8. Indicate the approximate number of myopic patients under 16 seen in a typical week:

1. ≤ 5 patients
2. 6-10 patients
3. 11-15 patients
4. 16-20 patients
5. > 20 patients

9. How concerned are you about the increasing frequency of paediatric myopia (onset between 5-16 years of age) in your practice?

|  |  |  |  |  |  |  |  |  |  |
| --- | --- | --- | --- | --- | --- | --- | --- | --- | --- |
| 1 | 2 | 3 | 4 | 5 | 6 | 7 | 8 | 9 | 10 |

10. To what extent do you think that the onset and progression of myopia is related to the incidence of ocular pathologies? (1-nothing related / 5-closely related):

|  |  |  |  |  |
| --- | --- | --- | --- | --- |
| 1 | 2 | 3 | 4 | 5 |

11. In your daily practice, do you apply any kind of treatment to try to slow the progression of myopia in your patients?

1. Yes
2. No

**II: Myopia management practice (only for optometrists undergoing treatment for myopia control)**

1. Which of the following clinical procedures do you routinely perform at the first visit of a school-age myope (5-16 years)? (You can select multiple options, if applicable)

|  | Family history of myopia |  | Ocular axial length measurement |
| --- | --- | --- | --- |
|  | Non-cycloplegic subjective refraction |  | Pupil size measurement |
|  | Cycloplegic subjective refraction |  | Undilated retinal fundus examination |
|  | Non-cycloplegic retinoscopy |  | Dilated retinal fundus examination |
|  | Cycloplegic retinoscopy |  | Optical coherence tomography (OCT) |
|  | Non-cycloplegic autorefraction |  | Retinography |
|  | Dynamic retinoscopy |  | Corneal topography |
|  | Cover test |  | Intraocular pressure |
|  | AC/A ratio |  | Stereopsis |
|  | Accommodative flexibility |  | Ocular motility |

2. Indicate your years of experience using treatments to control myopia progression:

1. I have no experience
2. ≤ 2 years
3. 3-5 years
4. 6-10 years
5. > 10 years

3. Do you think that this type of treatment can be more effective depending on the age at which it is applied?

1. Yes
2. No

4. How active would you consider your clinical practice in the field of myopia management? (1-not very active / 5-very active):

|  |  |  |  |  |
| --- | --- | --- | --- | --- |
| 1 | 2 | 3 | 4 | 5 |

5. Indicate the treatment you consider most effective for controlling the progression of myopia in a patient (5-16 years old) who is already myopic:

|  | Soft contact lenses (full correction) |  | Spectacles lenses for myopia control |
| --- | --- | --- | --- |
|  | Bifocal soft contact lenses |  | Bifocal spectacles lenses |
|  | Soft defocus contact lenses (myopia control) |  | Progressive addition spectacle lenses |
|  | RGP contact lenses |  | Orthokeratology |
|  | Bifocal RGP contact lenses |  | Atropine (no differentiation of concentrations) |
|  | Multifocal RGP contact lenses |  | Combination of Orthokeratology and Atropine |
|  | Spectacle lenses (full correction) |  | Visual hygiene |
|  | Spectacle lenses (under correction) |  | Increase time spent outdoors |

6. Indicate the type of recommendations you usually give to your myopic patients (5-16 years) (You can select several options):

|  | Spend more time outdoors |
| --- | --- |
|  | Decrease screens or television usage time |
|  | Decrease smartphone usage time |
|  | Read in daylight conditions whenever possible |
|  | Maintain an adequate reading distance |
|  | Vision therapy |
|  | None of the above |

7. From what age would you recommend each of the following options for a myopic patient?

|  | Never | < 5 years | 5-10 years | 11-15 years | 16-20 years |
| --- | --- | --- | --- | --- | --- |
| Single-vision spectacle |  |  |  |  |  |
| Bifocal spectacle lenses |  |  |  |  |  |
| Progressive spectacle lenses |  |  |  |  |  |
| Spectacle lenses for myopia control |  |  |  |  |  |
| RGP contact lenses |  |  |  |  |  |
| Soft defocus contact lenses (myopia control) |  |  |  |  |  |
| Orthokeratology |  |  |  |  |  |
| Pharmacological (must be prescribed by an ophthalmologist) |  |  |  |  |  |
| Refractive surgery |  |  |  |  |  |

8. Indicate the minimum amount of myopia (in dioptres) you consider for each of the following correction options for a myopic patient (age 5-16 years):

|  | Never | ≤ 1.00D | -1.25D to -3.00D | -3.25D to -5.00D | > -5.00D |
| --- | --- | --- | --- | --- | --- |
| Single-vision spectacle |  |  |  |  |  |
| Bifocal spectacle lenses |  |  |  |  |  |
| Progressive spectacle lenses |  |  |  |  |  |
| Spectacle lenses for myopia control |  |  |  |  |  |
| Single-vision RGP contact lenses |  |  |  |  |  |
| Soft defocus contact lenses (myopia control) |  |  |  |  |  |
| Orthokeratology |  |  |  |  |  |
| Pharmacological (must be prescribed by an ophthalmologist) |  |  |  |  |  |
| Refractive surgery |  |  |  |  |  |

9. What is the minimum level of myopia progression that you consider requires treatment for myopia control?

|  | -0.01 to -0.25 diopters |
| --- | --- |
|  | -0.26 to -0.50 diopters |
|  | -0.51 to -0.75 diopters |
|  | -0.75 to -1.00 diopters |
|  | <-1.00 diopters |

10. Indicate the relative importance of each of the following factors in deciding on treatment for a myopic child (age 5-16 years)

|  | Not relevant | Minor relevant | Moderately relevant | Relevant | Very relevant |
| --- | --- | --- | --- | --- | --- |
| Patient’s pupil size |  |  |  |  |  |
| Family socio-economic status |  |  |  |  |  |
| Patient’s ethnicity |  |  |  |  |  |
| Patient’s lag of accommodation |  |  |  |  |  |
| Patient’s near phoria |  |  |  |  |  |
| Refractive status of patient’s parents |  |  |  |  |  |
| Patient’s habitual working distance |  |  |  |  |  |
| Amount of time patient spends undertaking near work |  |  |  |  |  |
| Patient’s current age |  |  |  |  |  |
| Patient’s current refractive error |  |  |  |  |  |
| Patient’s rate of myopia progression in the past year |  |  |  |  |  |

11. Indicate the frequency with which you prescribe each of the following treatments/recommendations to myopic children (5-16 years)

|  | Never | Sometimes | About half of the time | Most of the time | Always |
| --- | --- | --- | --- | --- | --- |
| Bifocal lenses with horizontal prisms |  |  |  |  |  |
| Spectacle lenses (under correction) |  |  |  |  |  |
| Spectacles lenses for myopia control |  |  |  |  |  |
| Bifocal spectacles lenses |  |  |  |  |  |
| Progressive addition spectacle lenses |  |  |  |  |  |
| Spectacle lenses (full correction) |  |  |  |  |  |
| Soft defocus contact lenses (myopia control) |  |  |  |  |  |
| Orthokeratology |  |  |  |  |  |
| Soft contact lenses (full correction) |  |  |  |  |  |
| Atropine (High dose > 0.5%)* |  |  |  |  |  |
| Atropine (Moderate dose > 0.1%-0.5% <)* |  |  |  |  |  |
| Atropine (Low dose > 0.001%)* |  |  |  |  |  |
| Cyclopentolate (1.0%)* |  |  |  |  |  |
| Timolol* |  |  |  |  |  |
| Visual therapy |  |  |  |  |  |
| Eye exercises |  |  |  |  |  |
| Visual hygiene |  |  |  |  |  |
| Advise to increase time spent outdoors |  |  |  |  |  |

*Pharmacological treatment must be prescribed by an ophthalmologist

12. In your workplace, do you collaborate with an ophthalmologist who prescribes pharmacological treatment for myopia control?

1. Yes*
2. No

If yes, answer these 3 questions (Only for optometrists collaborating with an ophthalmologist who prescribes pharmacological treatments for myopia control).

a. What is the earliest age at which pharmacological drops are prescribed to treat myopia progression?

b. What type of eye drops are most commonly use? (Please select multiple options, if applicable)

|  | Atropine 0.01% |
| --- | --- |
|  | Atropine 0.5% |
|  | Atropine 1% |
|  | Pirenzepine |
|  | Ciclopentolate 1% |
|  | Tropicamide |
|  | Timolol |
|  | Other |

c. If a patient has discontinued treatment, has he/she experienced a rebound effect?

|  | Yes |
| --- | --- |
|  | No |
|  | None of my patients have discontinued their treatment |

13. Where you work, do you prescribe optometric (contact lenses or spectacles) treatment for myopia control?

1. Yes*
2. No

Is Yes, answer these 3 questions (Only for optometrists using optometric treatments for myopia control).

a. What is the youngest age at which you prescribe pharmacological drops to treat myopia progression?

b. What type of optometric treatment do you most commonly use? (Please select multiple options, if applicable)

|  | Soft contact lenses (full correction) |
| --- | --- |
|  | Bifocal soft contact lenses |
|  | Soft defocus contact lenses (myopia control) |
|  | RGP contact lenses |
|  | Bifocal RGP contact lenses |
|  | Multifocal RGP contact lenses |
|  | Orthokeratology |
|  | Spectacle lenses (full correction) |
|  | Spectacle lenses (under correction) |
|  | Spectacles lenses for myopia control |
|  | Bifocal spectacles lenses |
|  | Progressive addition spectacle lenses |

c. If you have discontinued treatment in a patient, did they experience a rebound effect?

|  | Yes |
| --- | --- |
|  | No |
|  | None of my patients have discontinued their treatment |

**III: Questions for practitioners who do not prescribe treatments for myopia control**

1. Indicate the relative importance of each of the following factors as a barrier limiting your ability to provide optimal clinical care for myopic children (age 5-16 years):

|  | Not relevant | Minor relevant | Moderately relevant | Relevant | Very relevant |
| --- | --- | --- | --- | --- | --- |
| Lack of interest in managing childhood myopia |  |  |  |  |  |
| Minimal financial incentive |  |  |  |  |  |
| Need to purchase additional clinical equipment |  |  |  |  |  |
| Lack of experience in providing clinical care to children with myopia |  |  |  |  |  |
| Insufficient support from workspace |  |  |  |  |  |
| Insufficient consultation time |  |  |  |  |  |
| Lack of high-quality evidence to confirm the safety of interventions |  |  |  |  |  |
| Lack of high-quality evidence to demonstrate efficacy of interventions |  |  |  |  |  |
| Absence of clinical guidelines to guide management |  |  |  |  |  |
| Lack of regulatory approval of interventions for slowing myopia progression |  |  |  |  |  |
| Insufficient time for professional development |  |  |  |  |  |
| Concern over medico-legal aspects of interventions other than spectacles |  |  |  |  |  |

2. Indicate the reason that has prevented the use of other treatments for myopia control if you have only adapted monofocal glasses/lenses to myopic patients:

|  | I do not believe that these treatments are effective |
| --- | --- |
|  | Results are not predictable |
|  | Lack of high-quality evidence to confirm the safety of interventions |
|  | The treatment price to the patient |
|  | Additional work time required for the professional |
|  | Lack of experience in providing clinical care to children with myopia |
|  | Risk/benefit ratio |
|  | Not enough patients with the required conditions to use other treatments |
|  | I am not allowed to perform this type of treatment in the work centre |
